# Lack of a synergistic effect of a non-viral ALS gene therapy based on BDNF and a TTC fusion molecule

**DOI:** 10.1186/1750-1172-6-10

**Published:** 2011-03-21

**Authors:** Ana C Calvo, María Moreno-Igoa, Renzo Mancuso, Raquel Manzano, Sara Oliván, María J Muñoz, Clara Penas, Pilar Zaragoza, Xavier Navarro, Rosario Osta

**Affiliations:** 1 Laboratorio de Genética Bioquímica (LAGENBIO-I3A), Facultad de Veterinaria, Instituto Aragonés de Ciencias de la Salud (I+CS), Universidad de Zaragoza. C/Miguel Servet, 177, 50013 Zaragoza, Spain; 2 Group of Neuroplasticity and Regeneration, Institute of Neurosciences, Department of Cell Biology, Physiology and Immunology, Universitat Autònoma de Barcelona, CIBERNED, 08193 Bellaterra, Spain

## Abstract

**Background:**

Amyotrophic lateral sclerosis (ALS) is one of the most devastating neurodegenerative diseases. Neurotrophic factors have been widely tested to counteract neurodegenerative conditions, despite their unspecific neuronal access. The non-toxic C-terminal fragment of the tetanus toxin (TTC) heavy chain has been studied not only as a carrier molecule to the CNS but also as a neuroprotective agent. Because the neurotrophic effects of BDNF have been demonstrated *in vitro *and *in vivo*, the question addressed in this work is whether a fusion molecule of BDNF-TTC may have a synergistic effect and enhance the neuroprotective properties of TTC alone in a mouse model of ALS.

**Methods:**

Recombinant plasmid constructs (pCMV-TTC and pCMV-BDNF-TTC) were injected into the quadriceps femoris and triceps brachialis muscles of SOD1^G93A ^transgenic mice at 8 weeks of age. The hanging wire and rotarod tests were performed to assess motor coordination, strength and balance. Electrophysiological tests, morphological assays of spinal cord sections of L2 and L4 segments, and gene and protein expression analyses were performed. The Kaplan-Meier survival analysis test was used for comparisons of survival. Multiple comparisons of data were analyzed using a one-way analysis of variance (ANOVA).

**Results:**

Treatment with the fusion-molecule BDNF-TTC and with TTC alone significantly delayed the onset of symptoms and functional deficits of SOD1^G93A ^mice. Muscle innervation was partially preserved with these treatments, and the number of surviving motoneurons in L2 spinal cord segment was increased particularly by the fusion protein induction. Inhibition of pro-apoptotic protein targets (caspase-3 and Bax) and significant phosphorylation of Akt and ERK were also found in the spinal cord of treated mice.

**Conclusions:**

Significant improvements in behavioral and electrophysiological results, motoneuron survival and anti-apoptotic/survival-activated pathways were observed with BDNF-TTC treatment. However, no synergistic effect was found for this fusion molecule. Although BDNF in the fusion molecule is capable of activating autocrine and neuroprotective pathways, TTC treatment alone yielded similar neuroprotection. Therefore, an accurate study of the neuroprotective effects of TTC fusion molecules should be performed to obtain a better understanding of its effects.

## Background

Amyotrophic lateral sclerosis (ALS) is one of the most devastating neurodegenerative diseases, involving the progressive loss of motoneurons, muscle weakness and atrophy. ALS occurs in 4 to 8 of every 100,000 individuals worldwide [1] and 10% of the affected people have an inherited genetic component [2]. Susceptibility to familial ALS (FALS) is associated with mutations in various genes, the most frequent being mutations in the superoxide dismutase-1 (SOD1) gene [3]. Transgenic mice over-expressing human SOD1 with a G93A mutation (SOD1^G93A^) are one of the best characterized experimental ALS models [4] and many preclinical trials have been conducted in these mice to test a wide range of potential therapeutic molecules [5].

The search for an efficient treatment that prevents motoneuronal death could be especially useful in ALS. The retrograde and trans-synaptic transport of the C-fragment of the tetanus toxin (TTC) into the central nervous system (CNS) after an intramuscular injection of naked DNA or recombinant protein has been widely demonstrated and was originally used for the transport of active molecules because of the carrier properties of TTC [6-8].

Interestingly, recent evidence has raised the potential use of the TTC heavy chain as a therapeutic agent for neurodegeneration. *In vitro *studies have reported that TTC rescues degenerating cultured neurons from apoptotic death through the activation of phosphatidylinositol 3-kinase (PI3K) and mitogen-activated protein kinase (MAPK) survival signaling pathways [9]. Furthermore, our group has recently described that the intramuscular gene delivery of TTC in SOD1^G93A ^mice exerts a positive effect on the prevention of neurodegeneration, improves motor function, inhibits apoptotic pathways and prolongs the survival of these animals [10].

The fusion of TTC and an active molecule, such as a trophic factor, could strengthen the neuroprotective properties of TTC. The combination of TTC- and glial-derived neurotrophic factor (GDNF) has been evaluated in a neonatal rat axotomy model [11] and in the ALS mouse model [12]. The combination of TTC with insulin growth factor (IGF-1) has also been assayed in transgenic SOD1^G93A ^mice [13], although the effect of TTC alone has not been compared in any of these studies. When the effect of TTC was compared to the fusion molecule *in vitro*, a significant increase in the survival capacity of neuronal cells was found [12]. However *in vivo*, no significant differences were observed, which is probably due to the possibility that the fusion molecule might follow a GDNF route and not the TTC route under axotomy conditions [11].

A previous study reported that some neurotrophic factors, in particular brain derived neurotrophic factor (BDNF), facilitate the internalization of TTC fusion molecules in motor nerve terminals [14]. We have also observed that TTC and the recombinant fusion protein BDNF-TTC inhibit apoptosis in cultured neurons, with the quimeric molecule being more effective than TTC alone [9]. BDNF may cause a relocalization of membrane domains containing TTC receptors by activating Trk receptors, thereby facilitating the neuronal internalization of TTC. This observation is supported by other authors who state that TTC activates intracellular pathways involving Trk receptors [15]. BDNF belongs to the family of neurotrophins and binds specifically to TrkB receptors to activate the intracellular signaling pathways that promote neuronal survival and the differentiation of neurons. The neurotrophic effects of BDNF on motoneuronal degeneration have been widely studied *in vitro *and *in vivo *[16,17]. This neurotrophin has also been proposed as a potential therapeutic agent for the treatment of human ALS [18], although no successful results have been achieved. This failure in the clinical application of BDNF may be due to the low efficacy of targeting the neurotrophic factor to motoneurons. TTC possesses a high affinity for motoneurons [6], and the fusion of BDNF to the TTC protein might increase its accessibility. Taking all of these observations together, we speculated that the fusion of BDNF to TTC might have a synergistic positive effect. In the present study, we treated transgenic SOD1^G93A ^mice intramuscularly with a plasmid encoding for the fusion protein BDNF-TTC and investigated whether the beneficial effects of TTC previously observed on SOD1^G93A ^mice were enhanced by the presence of BDNF.

## Methods

### Construction of recombinant plasmids carrying TTC and BDNF-TTC DNA

**Figure 1:**
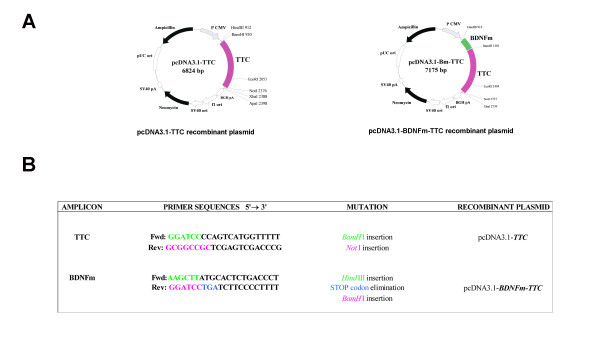
**Construction of pcDNA3.1-TTC and pcDNA3.1-BDNF-TTC recombinant plasmids**. **A**. Restriction maps of the recombinant plasmids pcDNA3.1-TTC and pcDNA3.1-BDNF-TTC. **B**. Primers used to amplify the sequences of TTC and of the mature form of BDNF were inserted in the recombinant plasmids of 6824 and 7175 base pairs, respectively.

Site-directed mutagenesis was used to introduce restriction sites of interest in the 5'/3' ends and/or to eliminate stop sequences to finally obtain the recombinant plasmids. The amplified sequences of TTC and BDNF were purified (QIAquick PCR purification kit, QIAGEN) and used as templates for cloning, using the PMOSBlue vector (Amersham Biosciences, GE Healthcare Europe GmbH, Barcelona, Spain), before being inserted in the eukaryotic expression plasmid. All potential therapeutic genes used in mice treatments were constructed in the pcDNA3.1 (Invitrogen S.A., Prat de Llobregat, Spain) eurkaryotic expression plasmid under the control of the cytomegalovirus (CMV) immediate-early promoter. pCMV:TTC was obtained by cloning a BamHI/NotI TTC fragment from the pGex:TTC vector [6] into pcDNA3.1. For pCMV:BDNF-TTC construction, the mature form of BDNF was obtained from a pcDNA3:BDNF vector (a gift from Dr. Pérez-Mediavilla, University of Navarra, Spain). PCR was used to eliminate the BDNF stop codon and to introduce HindIII (5'-end) and BamHI (3'-end) restriction sites for subcloning in the pMOSBlue vector. The HindIII/BamHI BDNF fragment was then introduced in pCMV:TTC to generate pCMV:BDNF-TTC (Figure 1). For the transformation assay, competent cells (*E. Coli DH5α *bacteria) were used, and the constructed plasmids were purified with QIAprep Spin Miniprep kit (QIAGEN). The sequence of the purified plasmids (BigDye Terminator v3.1 Cycle Sequencing kit, Applied Biosystems) was checked to confirm that the cloned DNA fragments were correctly inserted in the respective vectors. The recombinant plasmids were finally expanded in *E. Coli DH5α *bacteria and purified using the GenElute Endotoxin-free Maxiprep Plasmid Purification Kit (Sigma). This kit was suitable for gene therapy treatments due to its capacity to eliminate bacterial endotoxins during the plasmid extraction process. The recombinant plasmids were subjected to 1% agarose gel in 1X Tris-boric-EDTA (TBE) to yield fragments of the expected molecular weight. The quantity of the obtained recombinant plasmids was measured using a NanoDrop^® ^Spectrophotometer, ND-1000 V3.3.0, and plasmid concentration was adjusted to 1 μg/μl in a PBS solution.

### Transgenic mice

Transgenic mice with the G93A human SOD1 mutation (B6SJL-Tg[SOD1-G93A]1Gur) were purchased from The Jackson Laboratory (Bar Harbor, ME, USA). The colony was maintained by breeding male transgenic animals to naive (B6SJL) F1 females obtained from Charles River Laboratories (Belgium). All efforts were taken to minimize pain and discomfort and to maintain stable light/dark cycles.

The offspring were identified by PCR amplification of DNA extracted from tail tissue as described in the Jackson Laboratory protocol for the genotyping of hSOD1 transgenic mice http://jaxmice.jax.org/protocolsdb/f?p=116:2:351753648875652::NO:2:P2_MASTE R_PROTOCOL_ID,P2_JRS_CODE:523,002726 (based on [3]). Non-transgenic (wild-type) littermates were used as negative controls for the genetic background, and SOD1^G93A ^transgenic mice injected with empty plasmids were used as positive controls. Following the standard considerations for preclinical studies using SOD1 mice [19,20], all of the experimental designs were blinded and included the recommended number of animals. Equal numbers of balanced littermate males and females were assigned to the specific treatment groups.

Mice were housed in the Unidad Mixta de Investigación of the University of Zaragoza. Food and water were available *ad libitum*. All experimental procedures were approved by the Ethics Committees of our institutions and followed the guidelines for the use of laboratory animals based on the NIH Guide for Care and Use of Laboratory Animals (1985) and the European Communities Council Directive (86/609/EEC).

### In vivo intramuscular injections

Intramuscular injections of naked DNA encoding for pCMV-TTC, -BDNF, -BDNF-TTC and non-coding pCMV plasmids (50 μl, 1 μg/μl) were administered into the quadriceps femoris and the triceps brachialis muscles of balanced male and female SOD1^G93A ^transgenic mice at 8 weeks of age. Each quadriceps muscle was injected with 100 μg of plasmid in two injections, whereas each triceps muscle was given one injection with 50 μg of plasmid using an insulin syringe (25GA 5/8 Becton Dickinson SA, Madrid, Spain) following the guidelines for preclinical animal research [19,20]. A positive control group of SOD1^G93A ^transgenic mice was similarly injected with the same amount of empty plasmid.

For the PCR amplification for the detection of TTC expression in quadriceps extracts after the intramuscular injections of TTC and BDNF-TTC plasmids, the control positive group (i.e., the SOD1^G93A ^transgenic mice injected with empty plasmids) was used to test TTC expression in skeletal muscle. The negative control group (age-matched wild-type animals) was not injected with any of the recombinant plasmids. However, in relation to the western blot detection of TTC in spinal cord and skeletal muscle tissues, the negative control group and the positive control group were used to confirm that the detected bands corresponded to the recombinant protein but not to a technical error.

### Behavioral and functional evaluations

At two months of age, balanced males and females SOD1^G93A ^mice were blindly treated with the recombinant plasmids pCMVTTC, pCMV-BDNF, pCMV-BDNF-TTC or pCMV empty plasmid (positive control group) for behavioral analyses following the guidelines for preclinical animal research [19,20]. The control group, SOD1^G93A ^transgenic mice injected with empty plasmids, was used in this study to analyze the effect of naked DNA treatment during disease progression in ALS SOD1^G93A ^transgenic mice.

The onset of symptoms was evaluated using the rotarod test, which assesses muscle strength, motor coordination and balance [21,22]. All animals performed this test weekly beginning on the treatment day and followed the same methodology previously established in this animal model [21] according to the guidelines for preclinical animal research [19]. The hanging wire test was used to assess muscle strength. Each mouse was placed on the wire lid of a conventional housing cage. The lid was gently turned upside down 60-cm above a soft surface to avoid injuries. The latency to fall was timed. Each mouse was given up to three attempts to hold on to the inverted lid for a maximum of 180 s, and the longest period was recorded.

One week before the injections, the mice were trained daily on a Rotarod apparatus (ROTAROD/RS, LE8200, LSI-LETICA Scientific Instruments; Panlab S.L., Barcelona, Spain). After injections, SOD1 mutant mice were tested weekly for their ability to maintain their balance on the Rotarod apparatus at a constant speed of 15 rpm. The time an animal could remain on the rotating rod out of a maximum of 180 s was measured. The animals were tested three times during each session, and the best performance was recorded. Weight data were also recorded weekly from 8 weeks of age. Mice were sacrificed when they were unable to right themselves within 30 s after being placed on their side; this point was considered as the survival endpoint according to the guidelines for preclinical testing and colony management [19,20]. The weekly blinded monitoring for disease onset, disease progression and the end stage was in accordance to the guidelines for preclinical animal research [19], with the optimized method described in a previous work [21] and with similar studies in the same animal model [23]. The Kaplan-Meier test was used to analyze disease onset and life span.

### Electrophysiological tests and histological processing

Electrophysiological tests were performed at 3 and 4 months of age in other subgroups of SOD1^G93A ^treated as above, including negative (age-matched wild-type) and positive control (SOD1^G93A ^mice injected with empty plasmid) groups. Motor nerve conduction was evaluated by stimulating the sciatic nerve at the sciatic notch and recording the compound muscle action potentials (CMAP) from the gastrocnemius and plantar muscles with microneedle electrodes [24]. The evoked potentials were amplified and displayed on a digital oscilloscope to measure the amplitude from the baseline to the maximal negative peak and the latency from the stimulus to the onset of the first negative deflection. During electrophysiological tests, the animals were anesthetized (pentobarbital 40 mg/kg i.p.) and placed over a warm flat steamer to maintain body temperature that was monitored with a skin thermode. Following electrophysiological tests, the anesthetized animals were perfused with 4% paraformaldehyde in PBS at 4 months of age. The lumbar segment of the spinal cord was removed, post-fixed and cryopreserved in 30% sucrose. Transverse 40- μm-thick sections were serially cut using a cryotome. One section from each series of 10 sections was collected sequentially on gelatin-coated slides and stained with cresyl violet. The number of motoneurons present in both ventral horns was counted in four serial sections of each L2 and L4 spinal cord segments in all treated groups, and the negative and positive controls. Motoneurons were identified by their localization in the ventral horn and counted following strict size and morphological criteria [10,25]. The number of motoneurons in the treated groups was compared to the number of motoneurons in the negative (wild type) and positive (SOD1^G93A^) controls to study motoneuron survival under each treatment.

Other spinal cord sections of the L2 segment were blocked with TBS-Triton-FDS and incubated for 2 days at 4°C with primary antibody anti-BDNF (1:10000, Millipore) or anti-TTC (1:500, antibody SP48 purified in rabbit, Eurogentec, Belgium). After washes, sections were incubated for 1 day at 4°C with biotinilated secondary antibody (1:200; Vector) or with anti-rabbit Cy3-conjugated antibody (1:200, Jackson Immunoresearch). Finally, when necessary, they were incubated for 1 h with Cy3-conjugated streptavidin (1:1000, Zymed). Microphotographs of the grey matter of the ventral horn were taken at 200× and 400× to determine the immunolabeling of BDNF or TTC.

### Extraction of biological samples

Biological samples were extracted from SOD1^G93A ^transgenic mice and age-matched wild-type mice (negative control) that were anesthetized with a sodium pentobarbital solution (50 mg/kg i.p.) to extract the tissue samples as quickly as possible [26,27]. For the detection of injected plasmid expression, inoculated muscles were harvested 10 days after plasmid injections. Another group of naked DNA-treated (empty plasmid) SOD1^G93A ^transgenic mice (positive control) was sacrificed 50 days post-injections, and spinal cord tissues were harvested for gene expression and western blot analyses. Spinal cord tissue from non-transgenic age-matched mice was also extracted as negative controls for the genetic background.

### RNA extraction, synthesis of cDNA and PCR amplification from biological samples

Tissues were frozen in liquid nitrogen and stored at -70°C for subsequent RNA and protein extraction. For total RNA extraction, tissues were pulverized in a cold mortar using liquid nitrogen and homogenized with a pro200 homogenizer (PRO Scientific Inc., Oxford, CT, USA). TRIzol Reagent (Invitrogen S.A., Prat de Llobregat, Spain) was used for total RNA extraction from muscles according to the manufacturer's instructions. Spinal cord homogenized tissue from treated and positive (empty plasmid) control SOD1^G93A ^transgenic and wild-type mice was transferred to two pre-chilled tubes, one for total RNA extraction (RNeasy Mini Kit protocol, Qiagen-Izasa, Barcelona, Spain) and the other for protein extraction. RNA was treated to eliminate genomic DNA using Turbo DNA-free (Ambion, Madrid, Spain). For retrotranscription (RT), the SuperScriptTM First-Strand Synthesis System kit (Invitrogen S.A., Prat de Llobregat, Spain) was used for 1 μg of RNA in a final volume of 20 μl.

Intramuscular plasmid transfection and expression was detected with primers for TTC amplification in injected muscles. cDNA amplification reactions were performed in a final volume of 10 μl containing 300 nM of each primer (forward, 5'-ATGGAAGCAGTAAAATTGCGTGA-3' and reverse, 5'-TTGCCTATTTGACCATTATGGGTA-3'), 5 μl of 2X SYBR® Green PCR Master Mix (Applied Biosystems, Madrid, Spain) and 2 μl per reaction of 10× diluted cDNA for the detection of the TTC gene expression in muscle samples. All PCR reactions were performed using an ABI Prism 7000 Sequence Detection System (Applied Biosystems, Madrid, Spain). The thermal cycling parameters were as follows: incubation at 95°C for 10 minutes and 40 cycles of 95°C for 15 seconds and 60°C for 1 minute. The presence of the TTC gene amplicon (115 bp) was also observed in a 4% agarose gel stained with ethidium bromide.

Real-time PCR was used to study gene expression in the spinal cord from treated SOD1^G93A ^transgenic mice. The gene expression values were compared to the values obtained in the positive control group, whereas the negative control group was used as a reference to a non-pathologic situation. Reactions were performed in a final volume of 10 μl with a 1× TaqMan^® ^Universal PCR Master Mix (No AmpErase^® ^UNG, Applied Biosystems, Madrid, Spain), 1× of the primer and TaqMan^® ^MGB probe mix for each studied gene and 1 μl of a 10× -diluted cDNA per reaction. For normalization, 3 endogenous genes were used (18 S rRNA, GAPDH and β-actin). All reactions were performed in triplicate; the efficiency of the primer/probe sets was close to 100% in all reactions. Primer and probe mixtures for the genes of interest were supplied by Applied Biosystems (Madrid, Spain) (Table 1). PCR reactions were performed in an ABI Prism 7000 Sequence Detection System (Applied Biosystems, Madrid, Spain). The thermal cycling parameters were as follows: incubation at 95°C for 10 minutes, 40 cycles of and 95°C for 15 seconds and 60°C for 1 minute. The relative expressions of Gabra4 were normalized with the geometric mean of the three endogenous genes [28].

**Table 1:** Taqman^® ^probe and primer mixtures used in gene expression assays.

NAME	GENE SYMBOL	SEQUENCE	PROBE LOCATION	ORGANISM	PART NUMBER
gamma-aminobutyric acid (GABA-A) receptor	GABRA4	NM_010251.2	Exon 8-9	*Mus musculus*	Mm00802631_m1
subunit alpha 4					
glyceraldehyde-3-phosphate dehydrogenase	GAPDH	NM_008084.2	Exon 3	*Mus musculus*	4352932E
actin, beta, cytoplasmic	ACTB (β-actin)	NM_007393.1	Exon 6	*Mus musculus*	4352933E
18 S ribosomal RNA	18 S rRNA	X03205.1		*Homo sapiens*	Hs99999901_s1

GAPDH, β-actin and 18 S rRNA were used as housekeeping genes. Gabra4 was selected as a target gene to study GABAergic transmission under TTC, BDNF and BDNF-TTC treatments.

### Protein extraction and Western blot analysis

Powdered spinal cord tissue for protein extraction was resuspended in homogenization buffer [15,29,30] containing 150 mM NaCl, 50 mM Tris-HCl pH 7.5, 1% deoxycholate, 0.1% SDS, 1% Triton X-100, 1 mM Na_3_VO_4 _(a phosphatase inhibitor) together with the complete protease inhibitor cocktail 1 mM PMSF, 10 μg/ml leupeptin/aprotinin and 1 μg/ml pepstatin, and centrifuged for 10 min at 4°C at 3000 rpm. The supernatant was collected, and the protein concentration was quantified by BCA (Sigma-Aldrich Química, S.A., Madrid, Spain). Protein extracts (25 μg of total protein) were subjected to SDS/PAGE and transferred to PVDF membranes (Amersham Biosciences, GE Healthcare Europe GmbH, Barcelona, Spain) using a Mini Protean 3 (Bio-Rad, Hercules, CA, U.S.A.) at 100 V for 1 h. The blotting buffer used contained 25 mM Tris, 200 mM glycine and 10% (v/v) methanol. PVDF membranes were blocked for 1 h with Tris-buffered saline supplemented with 0.1% Tween 20 and 5% (w/v) skimmed powdered milk. The membranes were incubated overnight with the corresponding primary antibody diluted in blocking buffer: 1:500 anti-TTC, 50 KDa (antibody SP48 purified in rabbit, Eurogentec S.A., Belgium), 1:1000 Procaspase-3, 35 KDa (9662), 1:1000 p44/42 MAPK, 44/42 KDa (4348), 1:1000 phospho-p44/42 MAPK, 44/42 KDa (9106) (Cell Signaling Technology, Inc., Danvers, MA, USA); 1:200 Caspase-3, 11 KDa (AM65) (Calbiochem, San Diego, CA, USA); 1:1000 BDNF, ~ 20 KDa (sc-546), 1:2000 Bax, 23 KDa (sc-526), 1:2000 Bcl-2, 29 KDa (sc-492), 1:1000 total Akt, 60 KDa (sc-8312), 1:1000 phospho-Akt, 60 KDa (sc-7985-R), 1:5000 β-tubulin antibody, 55 KDa (sc-9104) (Santa Cruz Biotechnology, Inc., CA, USA). Several washes were performed with Tris-buffered saline/0.1% Tween 20 between each step. The secondary antibody was diluted 1:5000 in blocking buffer (goat anti-rabbit IgG-HRP or goat anti-mouse IgG-HRP, Santa Cruz Biotechnology, Inc.). The western blots were developed using Western Blotting Luminol Reagent (Santa Cruz Biotechnology, Inc., California 95060) and exposed to Agfa X-Ray films (Agfa, 2640 Mortsel, Belgium). The computer-assisted analysis of the bands was performed with AlphaEase FC software (Bonsai Technologies Group, S.A., Madrid, Spain). All the protein levels were tested in treated SOD1^G93A ^transgenic mice and compared to the positive (empty plasmid) control group and the levels observed in the negative control group, which served as a reference to a non-pathologic situation.

### Statistical analysis

All values are expressed as the mean ± S.E.M., and the n = number of mice in each group. Regarding the statistical analysis of the clinical outcomes, the Kaplan-Meier analysis was used for survival comparisons using a log-rank test to establish the efficacy of each treatment with a statistical significance set at a P value < 0.05. Multiple comparisons of data were analyzed using a one-way analysis of variance (ANOVA) followed by a Bonferroni post-hoc test. Variance in disease onset was analyzed using a one-way ANOVA followed by a Fisher's least significant difference (LSD) post-hoc test. Variances in motor function, strength and weight score curves were analyzed using a repeated measures ANOVA followed by Fisher's LSD testing. For gene expression and western blot data analysis, a one-way ANOVA was used followed by a Tukey post-hoc test. Statistical differences were considered significant at P < 0.05 level.

## Results

### Detection of TTC expression in the muscle and spinal cord of transfected SOD1^G93A ^mice

**Figure 2:**
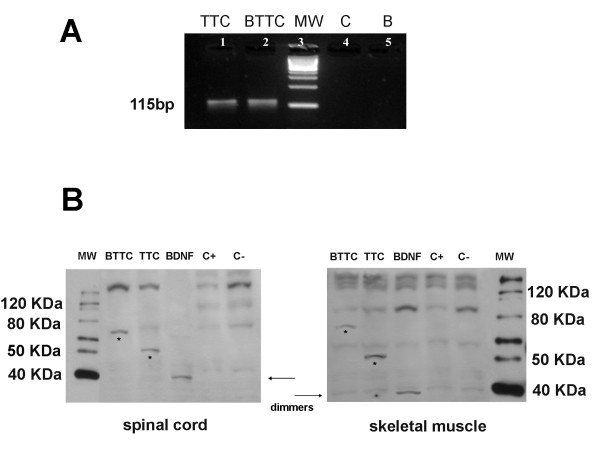
**TTC and BDNF detection in skeletal muscle and spinal cord of ALS transgenic SOD1^G93A ^mice**. **A**. PCR amplification for the detection of TTC expression in mouse quadriceps extracts after intramuscular injections of TTC and BDNF-TTC plasmids. (1) TTC plasmid injected quadriceps extract; (2) BDNF-TTC-plasmid injected quadriceps extract; (3) molecular weight marker, MW; (4) positive control, C (vehicle or empty-plasmid-injected quadriceps extract); (5) reaction blank (n = 5 mice per group). **B**. Western blot detection of TTC in spinal cord and skeletal muscle tissues of wild-type (C-, negative control), SOD1^G93A ^transgenic mice injected with empty plasmid (C+, positive control), TTC- and BDNF-TTC (BTTC)-treated mice. In TTC and BDNF-TTC treated groups, the detected band was approximately of 50 and ~ 70 KDa respectively (*), using both anti-TTC and anti-BDNF antibodies. In the BDNF group, the dimeric conformation, indicated by arrows, was observed at approximately 40 KDa (n = 5 mice per group).

We first confirmed the ability of the constructed vector to express the encoding gene within the muscle cells of SOD1^G93A ^transgenic mice. Because there is no endogenous expression of the TTC gene in the skeletal muscle of mice, PCR amplification of cDNA of the injected muscles was performed to detect mRNA expression of the gene. Previous studies on the spatial-temporal patterns of gene expression in mouse skeletal muscle after the injection of plasmid-DNA have revealed maximal expression levels between 7 and 14 days after injection [31]. The quadriceps muscles extracted from transgenic mice were used for the study of the expression of the plasmid constructs (pCMV-TTC and pCMV-BDNF-TTC) or empty plasmid 10 days after inoculation. As shown in Figure 2A, TTC gene expression was not observed in the control SOD1^G93A ^group (empty naked DNA-injected transgenic mice). In contrast, RT-PCR revealed the presence of the TTC gene amplification in muscles inoculated with the TTC and BDNF-TTC encoding vectors, indicating that they were transcribed in the muscle cells. Furthermore, to confirm that the TTC was transported to the spinal cord, a Western blot for TTC and BDNF proteins was performed. TTC and BDNF protein expression was only observed in the skeletal muscle and the spinal cord of TTC and BDNF-TTC-treated mice. BDNF was also detected in BDNF-treated mice. Conversely, neither TTC nor BDNF detection was found in wild-type and control SOD1^G93A ^mice (empty naked DNA-injected transgenic mice) (Figure 2B).

### Transport efficiency mediated by TTC

**Figure 3:**
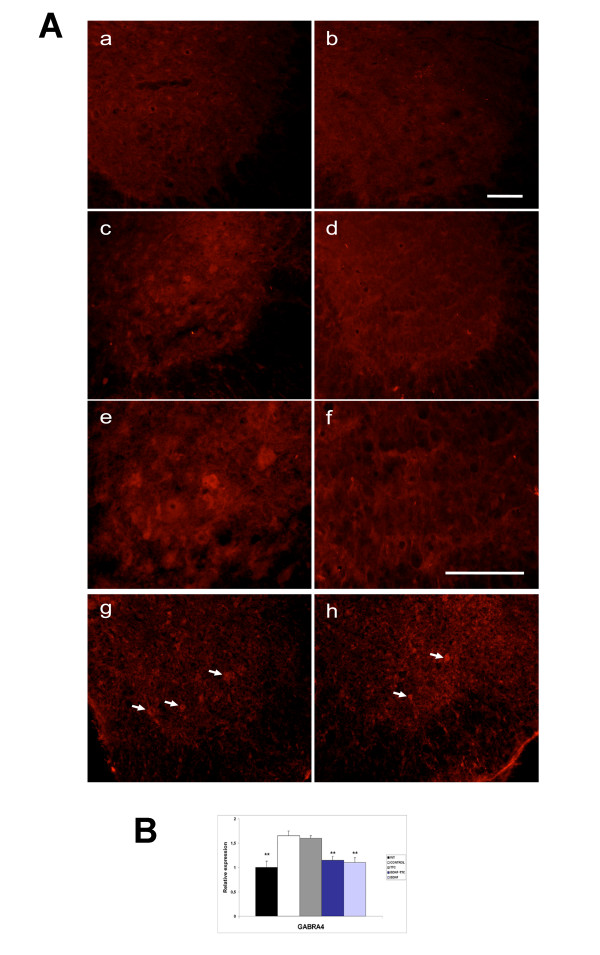
**Transport efficiency of BDNF-TTC molecule**. **A**. Immunohistochemical labeling for BDNF expression in the grey matter of the ventral horn of of (a) positive control (SOD1^G93A ^transgenic mice injected with empty plasmid), (b) SOD1^G93A^-BDNF and (c) L2 and (d) L4 spinal segments of SOD1^G93A^-BDNF-TTC mice. Note that BDNF is only expressed in the L2 spinal cord sections of mice treated with BDNF-TTC chimera and does not appear in other spinal cord segments. (e, f) Detail of BDNF immunolabeling of the sections shown in c and d, at higher magnification. Presence of TTC in the grey matter of the ventral horn of (g) SOD1^G93A^-BDNF-TTC and (h) SOD1^G93A^-TTC treated mice. Note more intense TTC labeling in animals treated with the chimera. Arrows point to some of the neurons positively stained for TTC. Bar = 200 μm in a, b, c, d, g and h; bar = 100 μm in e and f. **B**. Real-time PCR assay of a synaptic transmission-related gene in spinal cord specimens of symptomatic SOD1^G93A ^mice. Fold-changes in the expression of GABA(A) receptor subunit-4 (Gabra4) mRNA levels in total spinal cord of wild-type (negative control), positive control and treated transgenic mice. Results showed a significant down-regulation of the expression of this gene in neurotrophin-bearing treatments (BDNF-TTC and BDNF), approaching wild-type levels (*P < 0.05, **P < 0.01; error bars indicate SEM).

The efficiency of the transport of BDNF mediated by TTC was also detected immunohistochemically. The immunohistochemical labeling demonstrated positive BDNF and TTC immunoreactivity only in the soma of spinal motoneurons of mice treated with BDNF-TTC; spinal motoneurons of mice treated with BDNF did not show any labeling (Figure 3A). To confirm the long-term stability of the BDNF molecule, GABA(A) receptor gene expression was tested in the spinal cord samples of wild-type mice (negative control), positive control SOD1^G93A ^mice and BDNF-, BDNF-TTC- and TTC-treated mice. Real-time PCR was conducted on cDNAs obtained from total spinal cord extracts to examine whether treatment with the different plasmid constructs could affect GABA(A) receptor expression (subunit 4, Gabra4). In agreement with previous studies, our results showed a significant down-regulation of Gabra4 levels in BDNF-TTC- and BDNF-treated mice. However, TTC treatment did not affect the expression pattern of Gabra4 in the transgenic mice. Interestingly, a significant up-regulation of Gabra4 expression in the spinal cord of control SOD1^G93A ^mice was found compared to the wild-type age-matched animals (Figure 3B).

### Naked -DNA TTC and BDNF-TTC treatment improve disease clinical outcomes in SOD1^G93A ^mice

**Figure 4:**
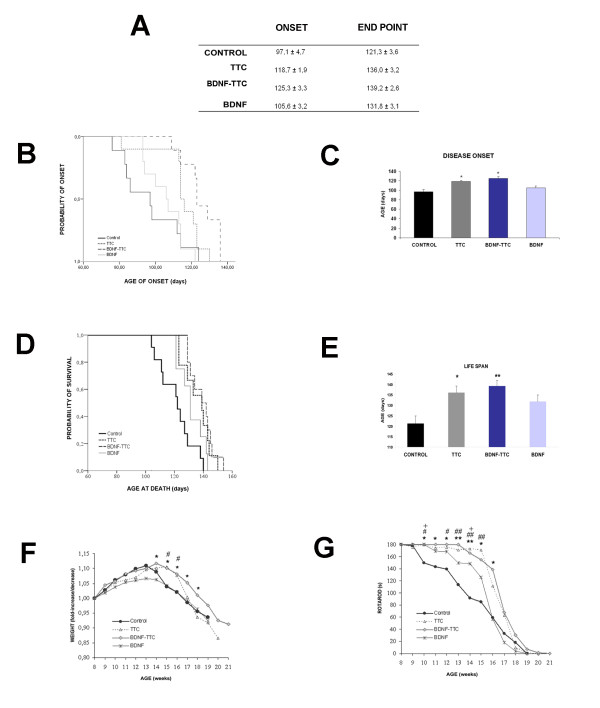
**Effect of naked -DNA treatment on the course of disease in ALS transgenic SOD1^G93A ^mice**. **A**. Table of the onset of symptoms and mortality of SOD1^G93A ^transgenic mice treated with naked DNA TTC, BDNF-TTC, BDNF and empty-plasmid (positive control) (n = 10 mice per group, balanced males and females). **B**. Cumulative probability of the onset of disease symptoms (hanging-wire test) and survival (**D**) in SOD1^G93A ^mice injected at 60 days of age with TTC, BDNF-TTC, BDNF or empty (positive control) plasmids. Bar graphs representing the average age of disease onset (**C**) and life span (**E**). **F**. Weight measurements of transgenic mice treated with the different constructions. **G **Strength and motor function were tested using the rotarod at 15 rpm. Mice were given up to 180 s for the test performance and the time at which mice fell was recorded (*, #, +, P < 0.05; **, ##, P < 0.01; error bars indicate SEM); * for BDNF-TTC vs. positive control comparisons; # for TTC vs. control comparisons; + for BDNF vs. positive control comparisons.

Due to the fact that the first signs of motor deficits in transgenic SOD1^G93A ^mice are usually detected near 100 days of age, naked DNA (TTC, BDNF-TTC and BDNF) treatment was administered at 8 weeks, which is well before the disease onset. The disease onset was mainly evaluated using the rotarod test. The intramuscular injection of pCMV-TTC or -BDNF-TTC plasmids delayed disease onset and improved motor function of SOD1^G93A ^treated mice compared to the positive control group. However, these improvements were not observed when pCMV-BDNF was injected (Figure 4). Significant differences in the TTC- and BDNF-TTC-treated groups (118.7 ± 1.9 and 125.3 ± 3.3 days, respectively) were found compared to the control group (97.1 ± 4.7 days) but not for BDNF-treated animals (105.6 ± 3.2 days) regarding the onset of the symptomatic phase (Figures 4A-C).

Regarding the life span, mice treated with naked DNA TTC (136.0 ± 3.2 days, log-rank test, p = 0.013) and with BDNF-TTC (139.2 ± 2.6 days, log-rank test, p = 0.001), but not mice treated with BDNF (131.8 ± 3.1 days, log-rank test, p = 0.175), showed significantly longer survival rates compared to the positive control SOD1^G93A ^mice (121.3 ± 3.6 days) (Figures 4D-E).

Animal weight has been demonstrated to be an objective parameter for disease progression in SOD1^G93A ^mice [21] with continuing weight gain in the pre-symptomatic phase (up to 13 weeks of age) and a denervation-induced weight loss in the symptomatic phase (Figure 4F). The inflection point of the weight curve for BDNF-TTC- and TTC-treated mice was delayed one and two weeks, respectively, but BDNF-treated animals did not differ from positive control SOD1^G93A ^mice. In addition, TTC treatment helped to maintain significantly higher weights in SOD1^G93A ^mice during 15 and 16 weeks of age, but BDNF-TTC treatment induced differences from 14 to 18 weeks of age, indicating a delay in disease progression.

The beneficial effect of naked DNA (TTC, BDNF-TTC and BDNF) treatment was also tested by an assessment of motor function and coordination on the rotarod from 8 weeks of age through the end-stage (Figure 4G). The rotarod performance during weeks 10 to 15 was significantly improved in TTC- and BDNF-TTC-treated mice compared to positive control SOD1^G93A ^mice. The latency on the rotarod began to decrease progressively after 10 weeks of age in control SOD1^G93A ^mice, whereas the transgenic mice transfected with TTC or BDNF-TTC plasmids showed a decrease of latency after 15 and 16 weeks of age, respectively. The overall rotarod performance was slightly improved in BDNF-treated mice, although no significant differences were observed with respect to the positive control SOD1^G93A ^mice except for two time points (10 and 14 weeks of age).

### Electrophysiological evaluation of the neuromuscular function of SOD1^G93A ^mice

**Figure 5:**
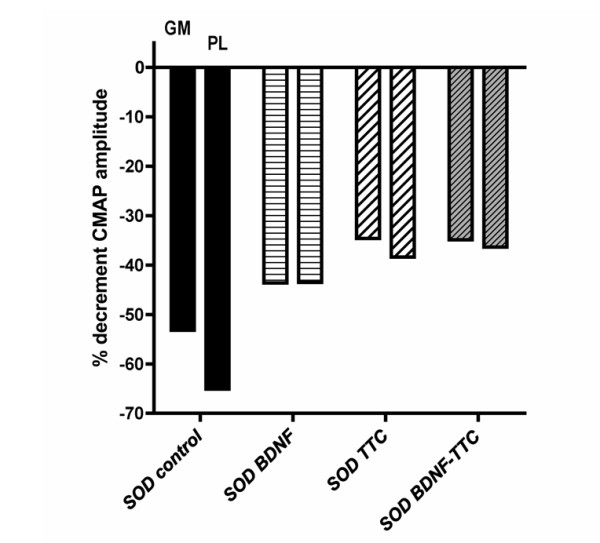
**Histogram representation of the decrement in the amplitude of the compound muscle action potential**. CMAP was compared at 4 months with respect to values at 3 months of age in SOD1^G93A ^mice, untreated and treated with naked DNA encoding for BDNF, TTC or BDNF-TTC. For each group, the left bar corresponds to the gastrocnemius muscle and the right bar to the plantar muscle (n = 7 mice per group).

The neuromuscular function of SOD1^G93A ^mice was assessed at 12 weeks, which is just before the time of clinical onset, and 16 weeks of age, which is during a late symptomatic stage. By 12 weeks of age, motor nerve conduction tests already revealed marked abnormalities, evidenced by a 40-60% decline in the amplitude and about a 20% increase in the latency of the CMAPs in the transgenic mice compared to age-matched wild-type mice (Table 2). From 3 to 4 months, there was a further marked reduction in the CMAP amplitudes in positive control SOD1^G93A ^mice to about 20-25% of normal values in plantar muscles and to about 45% in gastrocnemius muscles (Figure 5). This decline was less pronounced in BDNF-, TTC- and BDNF-TTC-treated mice (to 55%-65% of normal values), although these differences did not attain significance. These results agree with our previously published data that described *in vivo *that TTC treatment not only delayed the disease onset but also prolonged life span and improved coordination and motor/neuromuscular function [10].

**Table 2:** Neurophysiological study in gastrocnemius and plantar muscles.

Group	(n)	Gastrocnemius muscle		Plantar muscle	
		Latency (ms)	CMAP (mV)	Latency (ms)	CMAP (mV)
**3 months**					
**WT**	(6)	0.93 ± 0.04	51.9 ± 2.2	1.66 ± 0.08	6.49 ± 0.47
**SOD control**	(6)	1.11 ± 0.05	27.8 ± 2.9*	2.00 ± 0.10	2.55 ± 0.57*
**SOD BDNF**	(7)	1.12 ± 0.05	32.1 ± 5.0	1.89 ± 0.08	2.69 ± 0.44*
**SOD TTC**	(6)	1.04 ± 0.02	28.6 ± 3.5	1.95 ± 0.07	3.10 ± 0.52*
**SOD BDNF-TTC**	(8)	1.10 ± 0.05	32.1 ± 7.4	2.01 ± 0.11	2.64 ± 0.47*
**4 months**					
**WT**	(6)	0.93 ± 0.05	53.6 ± 4.2	1.59 ± 0.03	6.89 ± 0.79
**SOD control**	(5)	1.09 ± 0.04	13.0 ± 2.6*	1.98 ± 0.03	0.89 ± 0.35*
**SOD BDNF**	(5)	1.10 ± 0.06	18.1 ± 3.1*	1.88 ± 0.04	1.52 ± 0.41*
**SOD TTC**	(5)	1.06 ± 0.03	18.7 ± 4.8*	2.03 ± 0.15*	1.91 ± 0.83*
**SOD BDNF-TTC**	(5)	1.11 ± 0.07	20.9 ± 9.3*	2.02 ± 0.16*	1.68 ± 0.71*

Results of wild-type mice (WT), control SOD1^G93A ^mice, and SOD1^G93A ^mice treated with naked DNA encoding for BDNF, TTC, and BDNF-TTC are shown. Values are the mean ± SEM. CMAP, compound muscle action potential; n, number of mice.

* *P *< 0.05 vs. WT group at the same age.

### Spinal motoneuronal loss in SOD1^G93A ^mice

**Figure 6:**
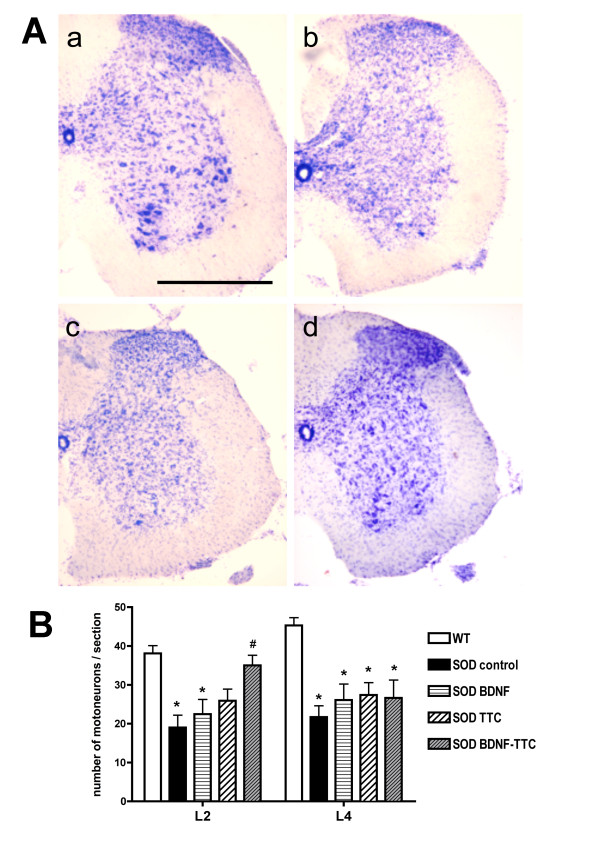
**Motor neuron survival in SOD1^G93A ^mice**. **A**. Representative micrographs showing cross-sections of lumbar spinal cords stained with cresyl violet from wild-type, **a**, SOD1^G93A ^control (positive control), **b**, BDNF-treated, **c**, and BDNF-TTC-treated, **d**, mice at 16 weeks of age. Bar = 500 μm. **B**. Histogram representation of the average number of stained motoneurons per section in L2 and L4 spinal cord segments of wild-type littermates, control SOD1^G93A ^and treated mice (n = 4-5 mice per group). * *p *< 0.05 vs. wild type; # *p *< 0.05 vs. SOD^G93A ^control mice.

The extent of motoneuronal degeneration was determined by counting the number of cresyl stained motoneurons in the ventral horns of lumbar spinal cord sections of SOD1^G93A ^mice at 16 weeks of age. Figure 6A shows representative spinal cord sections from wild-type, positive control SOD1^G93A ^mice and SOD1^G93A ^mice injected with pCMV-BDNF or pCMV-BDNF-TTC. The number of surviving motoneurons that met the size and shape criteria was significantly reduced in the lumbar spinal cord in SOD1^G93A ^mice compared to wild-type age-matched controls. Nevertheless, the group treated with BDNF-TTC showed a close to normal number of surviving motoneurons in the L2 segment, which corresponds to the quadriceps muscle motor nucleus, whereas the group treated with TTC had only moderate improvement. In sections corresponding to the L4 segment, the number of motoneurons was similar between the 3 treatment groups and was slightly higher than in the positive control SOD1^G93A ^mice (Figure 6B).

### Effect of naked -DNA treatment on apoptotic and survival pathways

**Figure 7:**
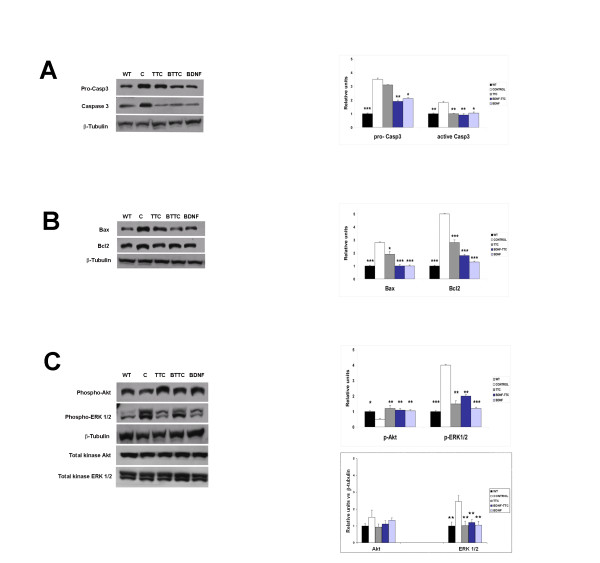
**Western blot analysis of proteins involved in the apoptotic and survival signalling pathways**. **A**. Fold-changes in the expression of pro-Casp3 and active Casp3 proteins. **B**. Bax and Bcl2 proteins and (**C**) phosphorylated states of Akt and ERK1/2 proteins in spinal cord lysates of control SOD1^G93A ^animals (white) and animals treated with TTC (grey), BDNF-TTC (blue, BTTC) and BDNF (soft blue). Western blot quantities are shown as the ratios to β-tubulin and then related to age-matched wild-type (black) mice data. Significant differences were found in all treated groups compared to control SOD1^G93A ^mice except for pro-Casp3 protein expression in TTC-treated mice. Biochemical behavior of treated transgenic animals resembled that of wild-type age-matched mice (n = 5 mice per group). The normalization of total protein ERK1/2 expression levels as a ratio to β-tubulin yielded a significant increase in the control group (white) with respect to the rest of groups (wild type, TTC-, BDNF- and BDNF-TTC-treated groups). The expression level of total protein of Akt and ERK 1/2 kinases was determined using the housekeeping protein β-tubulin (*P < 0.05 and **P < 0.01 vs. control SOD1^G93A ^mice; *** *p *< 0.001; error bars indicate SEM).

After intramuscular injections of the different plasmid constructions in SOD1^G93A ^mice, the spinal cord was analyzed at a late symptomatic stage to determine whether these molecules activated anti-apoptotic and survival pathways. The protein expression pattern of procaspase-3, activated caspase-3, Bax and Bcl2 proteins involved in apoptosis was studied. Mice treated with pCMV-TTC, BDNF-TTC or BDNF showed significantly lower levels of caspase-3 activation compared to the positive control SOD1^G93A ^mice. Caspase-3 activation levels were close to the levels observed in the spinal cords of wild-type age-matched mice, suggesting that the abnormal apoptotic process in SOD1^G93A ^mice was reduced by TTC, BDNF and BDNF-TTC treatment. Procaspase-3 protein levels were similar between the positive control group and the TTC-treated group, whereas mice treated with BDNF-TTC and BDNF showed a significant decrease in procaspase-3 expression patterns, approaching the levels of wild-type age-matched animals (Figure 7A).

Bax and Bcl2 have also been implicated in the degeneration of motoneurons in SOD1^G93A ^mice [32]. We found a significant upregulation in the expression of the pro-apoptotic protein Bax in the spinal cord of positive control SOD1^G93A ^mice relative to the wild-type age-matched mice (negative control). However, Bax levels approached normal levels in BDNF-TTC- and BDNF-treated animals. TTC-treated mice showed a slight but significant decrease in Bax expression compared to the positive control group. Regarding the anti-apoptotic Bcl2 protein, positive control SOD1^G93A ^mice had a significant increase in expression, but the different treatments caused a reduction in Bcl2 levels, indicating that these treatments could be restoring the neurodegenerative process by which Bcl2 expression is altered (Figure 7B). Because BDNF and TTC have already been described to be involved in the activation of 2 different survival signaling pathways *in vitro*, such as the PI3K/Akt and MAPK/ERK pathways [15], we investigated how treatment with the different plasmid constructs could affect these cascades in SOD1^G93A ^mice by analyzing the phosphorylation state of Akt and extracellular signal-regulated kinase (ERK) proteins in the spinal cord. We found that phospho-Akt levels were significantly higher in TTC-, BDNF-TTC- and BDNF-treated mice compared to positive control SOD1^G93A ^mice. Interestingly, phospho-Akt levels in all treated mice were similar to the levels of phospho-Akt in wild-type age-matched mice (negative control). However, phospho-ERK protein expression was increased almost 4-fold in positive control SOD1^G93A ^mice compared to the wild-type age-matched animals. In all treated animals, ERK activation levels were significantly reduced compared to positive control SOD1^G93A ^mice (Figure 7C). The expression levels of baseline Akt and ERK 1/2 kinases were also tested, and statistical significance was found only in baseline ERK 1/2 levels between the positive control group and the rest of groups (i.e., the negative control and TTC-, BDNF-TTC- and BDNF-positive-treated groups) in accordance with our previous results [22]. For this reason, Akt and ERK 1/2 phosphorylation levels were determined in relation to the housekeeping protein β-tubulin. The expression levels of both of the phosphorylated and baseline proteins in all of the treated animals, but not in the positive control group, showed a trend toward the normal levels of wild-type animals. These results suggest that the variations in the expression levels were due to the treatments.

## Discussion

The recombinant BDNF-TTC and GDNF-TTC proteins have shown a stronger effect than the TTC fragment alone in inhibiting apoptosis in cultured neurons [9,12]. Recently, *in vivo *studies [10] confirmed the therapeutic benefits of TTC in a murine model of motoneuron disease. Taking into consideration that the specificity of a trophic factor for motoneurons could be increased by genetically fusing it to TTC, while the trophic factor could contribute to enhance the benefits observed for TTC, our next approach was to test naked-DNA gene delivery to encode for a chimeric molecule, BDNF-TTC, in order to study the potential synergistic effect of these molecules in one of the best characterized neurodegenerative animal models, the SOD1^G93A ^mice.

Our results revealed that BDNF-TTC and TTC treatments induced a significant improvement in survival and motor function and a delay in the onset of symptoms and in the loss of body weight (Figure 4). Contrary to what we expected, no significant differences were found between the treatments. This fact suggests a lack of synergistic effect in the BDNF-TTC molecule and resembles other results with similar fusions [11], which point TTC as the main beneficial component of the fusion molecule. Therefore, we wondered whether the effect of BDNF-TTC treatment was mainly due to TTC because the recombinant plasmids were detected in skeletal muscle and their corresponding proteins reached the spinal cord tissue (Figures 2, 3A). This implied that BDNF in the fusion molecule was not active enough to exert a synergistic effect with TTC. Although the neuroprotective properties of other chimeric molecules, such as GDNF-TTC, have also been tested and yielded positive results [12], the genetic fusion of trophic factors to larger proteins could alter protein folding and conformation, thereby undermining the neuroprotective properties [33]. However, other authors have suggested that proteins coupled to TTC share its long-term stability [34]. Nevertheless, to assure this fact, the gene expression of the GABA(A) receptor was studied in the spinal cord tissue of all treated animals because the exposure to BDNF induces a reduction in postsynaptic GABA(A) receptor number in cultured neurons [35]. Our findings showed a downregulation of the expression of a GABA(A) receptor subunit in the spinal cord of BDNF- and BDNF-TTC-treated mice, but not in TTC-treated and control SOD1^G93A ^mice (Figure 3B), which suggests that BDNF influences GABA(A)-mediated transmission. These results also suggest that TTC might not exert its action at the inhibitory synaptic transmission level. However, GABA(A) receptor down-regulation may be a useful marker of the action of BDNF and can be used to confirm the active state of BDNF in the recombinant molecule.

The next step was to test whether the different efficacies of the injected molecules in reaching the spinal cord tissue could yield different responses depending on the treatment. To conduct this study, we tested motor and neuromuscular functions and motoneuronal survival in the treated mice and investigated the molecular pathways involved in the pathogenesis of ALS.

Despite being non-significant, we detected a reduction in the decline of CMAPs amplitude in all the treated animals compared to the control SOD1^G93A ^mice (Figure 5) and a higher number of motoneurons in L2 and L4 spinal cord segments in the treated animals than in the control SOD1^G93A ^mice. At L2, BDNF-TTC treated animals showed the highest number of surviving motoneurons, being higher than in TTC-treated animals, and close to the numbers of motoneurons in wild-type mice (Figure 6).

The main question that arises from these results is why this significant preservation of spinal motoneurons under BDNF-TTC treatment did not yield a significant response compared to TTC treatment in the survival and behavioral tests. One explanation could be the possible autocrine modulation of BDNF as observed in SOD1^G93A ^mice under iron-chelating drug treatment [36]. Similarly, because we confirmed the active state of BDNF in the recombinant molecule, this BDNF could activate survival pathways that promote the production of more BDNF through the activation of Akt and ERK [15].

An increase of phospho-Akt levels was found in treated animals compared to positive control SOD1^G93A ^mice and was similar to the expression level profiles under all of the treatments (Figure 7C). Although all of the treated groups showed lower levels of phospho-ERK than the positive control SOD1^G93A ^mice, which indicated a significant amelioration of motoneuronal degeneration in these animals, the highest phospho-ERK levels were detected with BDNF-TTC treatment (Figure 7C), suggesting that BDNF in the fusion molecule more efficiently reached the spinal cord where it may have exerted its autocrine effect. In fact, the TTC fragment contains a non-identified secretion signal peptide and, in the present study, we fused TTC to the mature form of BDNF that lacked its own signal peptide. Therefore, the fusion protein secretion must have followed the TTC secretion pathway, and this was confirmed by Western blot and by the immunohistochemical detection of BDNF in spinal cord samples of BDNF-TTC-treated mice (Figures 2B, 3A). Nevertheless, this BDNF-enhanced production through phospho-ERK activation was not enough to prompt a synergistic effect of the chimeric molecule.

The analysis of programmed cell death markers has provided evidence to suggest the implication of apoptotic pathways in ALS patients and in mouse models of the disease [37]. Our results showed a tendency to normalize the function of these pathways under all of the treatments administered. In particular, TTC, BDNF-TTC and BDNF treatments inhibited apoptotic pathways in the spinal cord of SOD1^G93A ^mice by reducing the activation of pro-caspase 3 and active caspase-3 and by reducing Bax and Bcl2 protein levels close to levels observed in wild-type mice (Figures 7A-B). Contrary to previous observations [32], Bcl2 over-expression was found in positive control SOD1^G93A ^mice. This might be due to a reactive up-regulation of Bcl2 expression because mutant SOD1 proteins bind and aggregate with Bcl2 in the spinal cord [38].

These results are in accordance with the well-known potential of BDNF to enhance the survival of motoneurons in culture and in *in vivo *models of degeneration [39], presumably through the activation of anti-apoptotic signals, and with the neuroprotective effects of TTC demonstrated *in vitro *and *in vivo*, including the prevention of the stress-induced death of cultured neurons and the activation of survival signaling pathways [9,10,15,40]. Interestingly, BDNF-TTC-treated animals showed a similar protein expression pattern to TTC-treated animals, especially of active caspase-3 levels. This suggests that TTC exploits a non-classical secretion pathway that mimics a neurotrophic secretion pathway but uses another route that might be important for the survival of motoneurons and prompting different biochemical activations in the spinal cord of SOD1^G93A ^mice. Therefore, BDNF-TTC, unlike BDNF alone, could also use the same pathway as TTC to induce a similar effect.

In summary, the first link in the destabilization chain of motor nerve terminals and the subsequent dying back of the axons and motoneurons remains unknown in ALS. The fact that TTC and BDNF-TTC can be transported through motoneurons to induce a later onset of symptoms, improve motoneuron survival and extend the survival of SOD1^G93A ^mice suggests that the naked DNA-mediated intramuscular delivery of TTC and BDNF-TTC fusion molecules promotes neuroprotective effects in the SOD1^G93A ^murine model of ALS. Nevertheless, our results suggest that this neuroprotective effect may be mainly due to TTC in the case of the fusion molecule, despite the active state of the trophic factor, and for this reason, testing the effects of TTC alone is a necessary step to tackle the potential effects of any fusion molecule based on TTC.

## Conclusions

We have studied the potential synergistic effect of the fusion molecule BDNF-TTC in SOD1^G93A ^mice, one of the best-characterized neurodegenerative animal models for ALS. Despite observing a significant improvement in behavioral, electrophysiological and immunohistochemical assays together with an activation of anti-apoptotic and survival pathways under BDNF-TTC treatment, no synergistic effect was found using the BDNF-TTC molecule. Moreover, the detection of BDNF-TTC in the spinal cord of SOD1^G93A^-treated mice and the active state of BDNF in the fusion molecule suggest that BDNF could exert an autocrine and neuroprotective role together with TTC to a similar extent as TTC alone, but this effect was not sufficient to enhance the survival signals observed under TTC treatment alone. A better understanding of the neuroprotective effects of the TTC fusion molecules needs to be studied carefully because the observed effects might be mainly due to TTC itself.

## Competing interests

The authors declare that they have no competing interests.

## Authors' contributions

MJM, PZ, XN and RO conceived the study, participated in its design and coordination and helped to draft the manuscript. ACC and MMI performed the research and helped to draft the manuscript. RM^2 ^and SA contributed to the therapeutic DNA construction and the behavioral tests. RM^1^, CP and XN performed the electrophysiological tests and immunohistological processing. All authors read and approved the final manuscript.
